# Utilization of SURGIFLO® Hemostatic Matrix Reduces Intraoperative Blood Loss During Posterior Instrumented Thoracolumbar Spinal Surgery for Patients With Adult Spinal Deformity

**DOI:** 10.7759/cureus.77478

**Published:** 2025-01-15

**Authors:** Hiroshi Takahashi, Toru Funayama, Hiroshi Noguchi, Kousei Miura, Kentaro Mataki, Yosuke Shibao, Fumihiko Eto, Kosuke Sato, Tomoyuki Asada, Hisanori Gamada, Kento Inomata, Shun Okuwaki, Kotaro Sakashita, Tomoaki Shimizu, Takahiro Sunami, Masashi Yamazaki, Masao Koda

**Affiliations:** 1 Department of Orthopaedic Surgery, Institute of Medicine, University of Tsukuba, Tsukuba, JPN; 2 Department of Orthopaedic Surgery, Tokyo Medical University Ibaraki Medical Center, Ami, JPN; 3 Department of Orthopaedic Surgery, Ibaraki Western Medical Center, Chikusei, JPN; 4 Department of Orthopaedic Surgery, Mito Medical Center, Mito, JPN; 5 Department of Orthopaedic Surgery, Kenpoku Medical Center/Takahagi Kyodo Hospital, Takahagi, JPN; 6 Department of Orthopedic Surgery, Hospital for Special Surgery, New York, USA

**Keywords:** adult spinal deformity, blood transfusion, instrumented thoracolumbar spinal surgery, surgical treatment, surgiflo hemostatic matrix

## Abstract

Background

Massive blood loss is a major complication of posterior instrumented thoracolumbar surgery for adult spinal deformities. The use of flowable and absorbable hemostatic products, such as SURGIFLO^®^ Hemostatic Matrix (SURGIFLO; ETHICON, Inc., Raritan, NJ, US), has become feasible for spinal surgery. Hence, a retrospective observational study was conducted to evaluate the efficacy of SURGIFLO during posterior instrumented thoracolumbar spinal surgery for an adult spinal deformity.

Methods

This analysis included 36 consecutive patients who underwent posterior instrumented thoracolumbar spinal surgery for an adult spinal deformity at our hospital from 2018 to 2022. Patients were divided into two groups: 19 who received SURGIFLO after March 2020 (Group S) and 17 who did not receive SURGIFLO before March 2020 (Group C). Operation time, intraoperative blood loss, postoperative blood loss (calculated from the suction drain until the next morning), and the number of perioperative blood transfusions were investigated.

Results

No significant differences were found between groups in terms of the number of fusion levels, osteotomy grades, and average operative times. However, Group S showed significantly less intraoperative blood loss per intervertebral level compared with Group C (p=0.023). Although the amount of concentrated red cells used did not differ significantly between groups, Group S required significantly less perioperative fresh frozen plasma than Group C (p=0.021).

Conclusion

In patients with an adult spinal deformity undergoing posterior instrumented spinal surgery from the thorax to the pelvis, utilization of SURGIFLO Hemostatic Matrix reduced intraoperative blood loss and the perioperative use of fresh frozen plasma.

## Introduction

With the progressive aging of society in recent years, the incidence of adult spinal deformity (ASD) has been increasing. ASD can sometimes impede activities of daily living (ADL) and quality of life (QOL) due to reduced mobility caused by severe low back pain and loss of muscle volume [1]. Recent reports have indicated that surgical intervention is more effective than conservative treatment for ASD, both in terms of clinical outcomes and cost-effectiveness [2,3]. Consequently, surgical treatment is generally recommended for patients with ASD. However, it is widely acknowledged that surgical intervention for ASD presents significant challenges for both surgeons and patients due to the necessity of exposing numerous spinal levels from the thorax to the pelvis and performing multilevel osteotomies for sagittal correction [4]. A recent report indicated that the incidences of the complications of massive bleeding, neurological deficit, and instrumentation failure are 8.01%, 3.07%, and 5.21%, respectively [5]. Notably, massive bleeding is a major and critical complication. To mitigate this complication, various advances have been made in recent years. SURGIFLO^®^ (SURGIFLO; ETHICON, Inc., Raritan, NJ, US) is a flowable and absorbable hemostatic product by mixing a gelatin matrix and a thrombin solution [6]. It was approved for use in spinal surgery in Japan in 2019. Since March 2020, this hemostatic matrix has been used to mitigate massive bleeding during posterior instrumented surgery for ASD. However, its efficacy in preventing blood loss for this type of surgery remains uncertain. Therefore, a retrospective observational study to assess the efficacy of SURGIFLO during posterior instrumented thoracolumbar spinal surgery for ASD, as well as to compare surgical outcomes before and after its implementation at our institution, was conducted.

## Materials and methods

Patient population

This study is a retrospective observational study to assess the efficacy of SURGIFLO during posterior instrumented thoracolumbar spinal surgery for ASD, as well as to compare surgical outcomes before and after its implementation at our institution. This study was approved by the Institutional Review Board of Tsukuba Clinical Research & Development Organization (R06-110). Informed consent for participation and the use of data was obtained from all the patients. This consent was obtained in accordance with the ethical standards of the institutional and national research committee and with the 1964 Helsinki Declaration and its later amendments. For this retrospective study, informed consent for publication was obtained through publicly available documents, as the study involved anonymized data and posed minimal risk to participants. This approach was approved by the Institutional Review Board. From 2018 to 2022, 36 consecutive patients who underwent posterior instrumented spinal surgery from the thoracic vertebra to the pelvis for ASD at our hospital were included. Patients who underwent single-stage combined anterior and posterior surgery were excluded. The patients were divided into two groups: 19 patients who received SURGIFLO after March 2020 (Group S) and 17 patients who did not receive SURGIFLO before March 2020 (Group C). In all patients, a microfiber collagen hemostat (Avitene^TM^; Becton, Dickinson and Company, Warwick, RI, US) was used as a standard practice.

Surgical procedure

**Figure 1:**
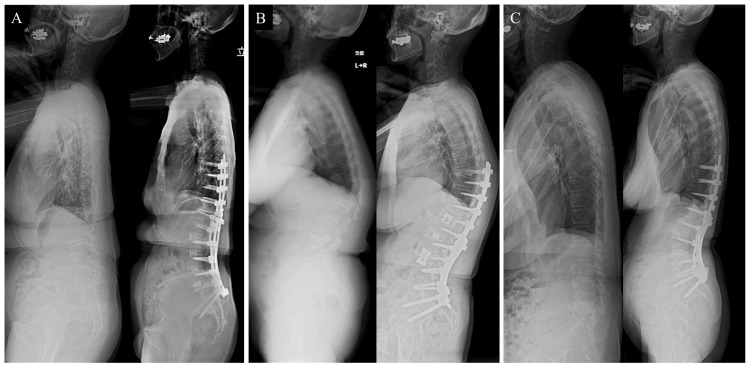
Lateral X-ray images of the representative cases before and after surgery (A) First-stage lateral lumbar interbody fusion was performed from L2/3, L3/4, and L4/5. One week later, second-stage posterior corrective instrumented fusion surgery was performed from T9 to the pelvis. (B) First-stage posterior corrective instrumented fusion surgery was performed from T8 to the pelvis. One week later, second-stage anterior cage insertion with lateral interbody fusion was performed. (C) First-stage L2/3 lateral lumbar interbody fusion was performed. One week later, second-stage L4 pedicle subtraction osteotomy (grade 3) and posterior corrective fusion surgery from T10 to the pelvis were performed.

All operative procedures were performed by senior spinal surgeons at a single institution. As a standard practice for ASD surgery planning, the flexibility of the lumbar spine was assessed with an X-ray in the fulcrum backward bending position [7]. When the patient exhibited flexibility, a two-stage surgery was planned. In the first stage, lateral interbody fusion (LIF) was performed. Approximately one week later, posterior instrumented fusion surgery with a posterior-column osteotomy (grade 2) from the thorax to the pelvis was conducted as the second-stage surgery (Figure 1A). For patients with osteoporotic vertebral fractures and dynamic instability, posterior instrumented corrective fusion was performed in the first stage, followed by anterior column replacement in the second stage (Figure 1B). For patients with limited flexibility, pedicle subtraction osteotomy (grade 3) was conducted via a two-stage surgery (Figure 1C). In the present study, the operative time and the amount of bleeding in anterior surgery were excluded, and the focus was solely on the measured values from posterior surgery.

Use of SURGIFLO

**Figure 2:**
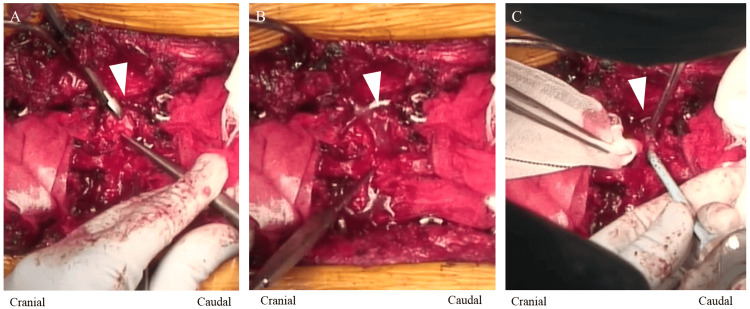
Actual use of SURGIFLO during surgery (A) Resection of the L4 upper facet of the right side. (B) After L4 upper facetectomy, bleeding from the epidural venous plexus was observed. (C) SURGIFLO is applied to stop the bleeding from the epidural venous plexus.

During surgery, SURGIFLO was utilized primarily to manage bleeding from the epidural venous plexus. Specifically, it was applied to control bleeding that occurred after upper facetectomy in the grade 2 osteotomy lesion (Figure 2).

Outcome measures

First, the operation time, intraoperative blood loss, and postoperative blood loss (calculated from the suction drain until the following morning) were assessed. These measured values were then calculated per intervertebral level. Second, the perioperative blood transfusions, including concentrated red cells (CRC) and fresh frozen plasma (FFP), were examined. Third, laboratory data, including preoperative and postoperative day 1 hemoglobin and platelet levels, were analyzed. Finally, the incidence of adverse events associated with the use of SURGIFLO in Group S was examined.

Statistical analyses

Results are presented as mean ± standard deviation. A student's t-test and a chi-square test were used to compare the relationship of each measured value between the two groups. P < 0.05 was considered significant in the tests of statistical inference. All statistical analyses were performed using the JMP software package (version 14.2.0; SAS Institute, Cary, NC, USA).

## Results

Patient characteristics

Patient characteristics are shown in Table 1. Group S had a significantly higher proportion of elderly individuals and females compared with Group C. The lower instrumented vertebra was the pelvis, and S2 alar iliac screws were inserted in all patients. There were no significant differences in body mass index (BMI), American Society of Anesthesiologists Physical Status (ASA-PS), levels of the upper instrumented vertebra (UIV), the number of fusion levels, and osteotomy grade.

**Table 1:** Patient characteristics BMI: Body Mass Index, ASA-PS: American Society of Anesthesiologists Physical Status, UIV: Upper Instrumented Vertebra

	Group S (n=17)	Group C (n=19)	P	t	χ2
Age	72.4 ± 5.9 (53-78)	67.3 ± 8.5 (50-82)	0.045	2.081	-
Sex (male/female)	1/16	7/12	0.031	-	4.976
BMI	23.5 ± 5.7	24.1 ± 2.7	0.733	0.344	-
ASA-PS 1	1	2	0.766	-	0.533
PS 2	9	8	-	-	-
PS 3	7	9	-	-	-
UIV T3	2	0	0.234	-	6.827
T4	2	0	-	-	-
T6	1	5	-	-	-
T8	1	1	-	-	-
T9	4	4	-	-	-
T10	7	9	-	-	-
Number of fusion levels	11.1 ± 2.7 (9-16)	10.4 ± 1.7 (9-13)	0.322	1.003	-
Osteotomy grade-None	0	1	0.439	-	2.707
Grade 1	0	1	-	-	-
Grade 2	15	13	-	-	-
Grade 3	2	4	-	-	-

Operation time, blood loss, and perioperative blood transfusion

Operation time, blood loss, and perioperative blood transfusion during surgery in each group are shown in Table 2. There was no difference between groups in operation time. Group S showed significantly less intraoperative blood loss per intervertebral level compared with Group C. However, there was no significant difference in postoperative blood loss between groups. In addition, significantly less perioperative FFP was used for Group S.

**Table 2:** Operation time, blood loss, and perioperative blood transfusion CRC: Concentrated Red Cells; FFP: Fresh Frozen Plasma

		Group S	Group C	P	t
Operation time (min)	Total	470 ± 104	505 ± 87	0.27	-1.122
-	Per intervertebral	44 ± 12	49 ± 9.3	0.13	-1.555
Intraoperative blood loss (g)	Total	1243 ± 634	1900 ± 1379	0.081	-1.798
-	Per intervertebral	109 ± 45	182 ± 117	0.023	-2.383
Postoperatibve blood loss (g)	Total	407 ± 177	371 ± 195	0.57	0.576
-	Per intervertebral	38 ± 17	37 ± 19	0.86	0.180
The amount of perioperative blood transfusion (unit)	CRC	3.8 ± 2.2	5.5 ± 3.6	0.10	-1.674
-	FFP	1.9 ± 2.3	4.6 ± 4.2	0.021	-2.412

Laboratory data

Changes in laboratory data are shown in Table 3. There are no significant differences in the change in hemoglobin or platelets between the two groups.

**Table 3:** Laboratory data

		Group S	Group C	P	F
Hemoglobin (g/dL)	Preoperative	11.2 ± 1.1	11.3 ± 1.7	0.163	2.035
-	Postoperative day 1	9.5 ± 1.2	10.4 ± 1.2	-	-
Platelet (10^3^/µL)	Preoperative	307 ± 137	319 ± 105	0.598	0.283
-	Postoperative day 1	187 ± 70	207 ± 81	-	-

Adverse events

Two of the 19 patients in group C experienced complications with surgical site infection and required additional wash and debridement surgery. However, the incidence was not statistically different between the two groups. There were no other adverse events associated with the use of SURGIFLO.

## Discussion

Reports on the utilization of SURGIFLO in spinal surgery are occasionally encountered [6,8,9]. However, they focus predominantly on relatively less invasive lumbar spinal surgeries such as laminectomy or short-segment posterior lumbar interbody fusion. Thus, the present study is the first to evaluate the impact of using SURGIFLO on surgical outcomes of posterior instrumented fusion from the thorax to the pelvis for patients with ASD. According to a recent report on posterior surgery for lumbar degenerative disorders, SURGIFLO was found to reduce blood loss during the hemostatic process and postoperative drainage volume compared with an absorbable gelatin sponge [6]. In the present study, although SURGIFLO decreased the intraoperative blood loss per intervertebral level and the amount of perioperative FFP used, it did not reduce postoperative blood loss. The authors speculate that SURGIFLO is often depleted before wound closure in ASD surgery due to the necessity of multilevel osteotomy, which may explain this phenomenon.

In the present study, no severe adverse events related to the use of SURGIFLO were observed. However, anaphylactic shock was reported to occur after SURGIFLO usage during posterior instrumented spinal surgery in a patient with adolescent idiopathic scoliosis [10]. In that case report, SURGIFLO was employed to reduce blood loss from the screw hole during pedicle screw insertion. In the present series, such severe adverse events did not occur, as SURGIFLO was used primarily to address bleeding from the epidural venous plexus during osteotomy. Nevertheless, caution should be exercised when using SURGIFLO for bleeding from cancellous bone.

The present study has several limitations. First, it had a small sample size and was a retrospective observational study conducted at a single institution, rather than a prospective multicenter study. However, the detailed surgical procedure is more standardized in a single institution study compared with those in multicenter studies, which may represent a strength. Nevertheless, to validate the findings of the present study, a prospective multicenter study with a larger sample size is warranted. Second, there was some bias in the degree of surgical invasion, such as the number of fused segments and the grade of osteotomy, in this study. To mitigate this bias, a study in a larger population that includes standardized surgical invasion criteria is necessary. Third, the present study lacks an evaluation of the patient's clinical symptoms, such as low back pain, using a visual analog scale or Oswestry Disability Index. However, regarding surgical outcomes for ASD, improvement in low back pain due to sagittal imbalance occurs when adequate correction of sagittal alignment is achieved [7]. The main focus of the present study is to evaluate the surgical invasion related to bleeding. Hence, clinical outcomes including low back pain were not evaluated. Fourth, the present study assessed only the use of SURGIFLO and lacked comparisons of SURGIFLO and other hemostatic matrices such as FLOSEAL (Baxter Healthcare Corporation, Hayward, CA, US) or tranexamic acid. A recent report indicated that tranexamic acid is useful for decreasing bleeding in ASD surgery [11]. The mechanism and route of administration of tranexamic acid differ from those of SURGIFLO, suggesting that combination therapy may be beneficial in preventing massive bleeding. On the other hand, FLOSEAL, similar to SURGIFLO in mechanism and route of administration, was reported to have no significant difference in estimated blood loss and blood transfusion rates compared with SURGIFLO [8,12]. Therefore, similar results may be expected with FLOSEAL instead of SURGIFLO. Finally, the use of SURGIFLO incurs additional costs. However, a recent report indicated that the use of SURGIFLO provides cost advantages. The authors believe that employing SURGIFLO is effective in preventing massive bleeding and is a cost-effective option.

## Conclusions

In patients with ASD undergoing posterior instrumented spinal surgery from the thorax to the pelvis, the use of SURGIFLO Hemostatic Matrix reduced intraoperative blood loss and perioperative use of FFP. These findings highlight the potential role of SURGIFLO as an adjunct to reduce surgical complications associated with excessive bleeding, thereby improving perioperative management strategies. Further prospective multicenter studies with a larger population are necessary to enhance the level of evidence for the efficacy of SURGIFLO in this application. In addition, comparisons with other hemostatic agents and combination therapies should be explored to optimize bleeding control during instrumented spinal surgeries.
